# Differences and similarities in selenium biopathways in *Astragalus, Neptunia* (Fabaceae) and *Stanleya* (Brassicaceae) hyperaccumulators

**DOI:** 10.1093/aob/mcad110

**Published:** 2023-08-21

**Authors:** Antony van der Ent, Mirko Salinitro, Dennis Brueckner, Kathryn M Spiers, Sofia Montanari, Annalisa Tassoni, Michela Schiavon

**Affiliations:** Laboratory of Genetics, Wageningen University and Research, Wageningen, The Netherlands; Centre for Mined Land Rehabilitation, Sustainable Minerals Institute, The University of Queensland, Brisbane, Queensland, Australia; Université de Lorraine, INRAE, LSE, F-54000 Nancy, France; Department of Biological Geological and Environmental Sciences, University of Bologna, Bologna, Italy; Deutsches Elektronen-Synchrotron DESY, Hamburg, Germany and; Deutsches Elektronen-Synchrotron DESY, Hamburg, Germany and; Department of Biological Geological and Environmental Sciences, University of Bologna, Bologna, Italy; Department of Biological Geological and Environmental Sciences, University of Bologna, Bologna, Italy; Department of Agricultural, Forest and Food Sciences (DISAFA), University of Turin, Turin, Italy

**Keywords:** synchrotron-based X-ray fluorescence microscopy, histology, light microscopy, X-ray fluorescence microscopy computed tomography, selenium two-dimensional mapping

## Abstract

**Background and Aims:**

Selenium hyperaccumulator species are of primary interest for studying the evolution of hyperaccumulation and for use in biofortification because selenium is an essential element in human nutrition. In this study, we aimed to determine whether the distributions of selenium in the three most studied hyperaccumulating taxa (*Astragalus bisulcatus*, *Stanleya pinnata* and *Neptunia amplexicaulis*) are similar or contrasting, in order to infer the underlying physiological mechanisms.

**Methods:**

This study used synchrotron-based micro-X-ray fluorescence (µXRF) techniques to visualize the distribution of selenium and other elements in fresh hydrated plant tissues of *A. racemosus*, *S. pinnata* and *N. amplexicaulis*.

**Key Results:**

Selenium distribution differed widely in the three species: in the leaves of *A. racemosus* and *N. amplexicaulis* selenium was mainly concentrated in the pulvini, whereas in *S. pinnata* it was primarilylocalized in the leaf margins. In the roots and stems of all three species, selenium was absent in xylem cells, whereas it was particularly concentrated in the pith rays of *S. pinnata* and in the phloem cells of *A. racemosus* and *N. amplexicaulis*.

**Conclusions:**

This study shows that *Astragalus*, *Stanleya* and *Neptunia* have different selenium-handling physiologies, with different mechanisms for translocation and storage of excess selenium. Important dissimilarities among the three analysed species suggest that selenium hyperaccumulation has probably evolved multiple times over under similar environmental pressures in the US and Australia.

## INTRODUCTION

Hyperaccumulators are plants that have the ability to accumulate specific metals and metalloids from the soil to extremely high concentrations in their shoot tissues, typically at concentrations 100- to 1000-fold higher than those found in non-hyperaccumulating species (van der Ent *et al.*, 2013). The term ‘hyperaccumulator’ was first used to describe plants with >1000 mg kg^−1^ nickel (Ni) in dry leaves (Brooks *et al.*, 1977), but it was then extended to other elements besides Ni (van der Ent *et al.*, 2013). To date, >700 species have been documented to hyperaccumulate one or more metal/metalloid elements, including arsenic (As), cadmium (Cd), cobalt (Co), copper (Cu), manganese (Mn), selenium (Se), zinc (Zn) and rare earth elements (van der Ent *et al.*, 2013; Reeves *et al.*, 2018). Most hyperaccumulating species in temperate regions are flowering plants of the Brassicaceae and Asteraceae families (Cappa and Pilon-Smits, 2014), whilst in tropical regions they are mainly members of the Phyllanthaceae, Proteaceae and Myrtaceae families (Reeves *et al.*, 2018).

Selenium hyperaccumulators, plants that accumulate Se in concentrations >1000 μg Se g^−1^ in natural environments, were first discovered in parts of the central western USA characterized by Se-enriched soils, the so-called seleniferous soils (Beath *et al.*, 1940) derived from particular Cretaceous shale rocks. Typical concentrations of Se in soils are on average 0.01–2 μg g^−1^, but in seleniferous soils the values can greatly exceed 100 μg g^−1^ (White, 2016). Seleniferous soils host not only hyperaccumulator species, but also plant species classified as secondary Se accumulators (100–1000 μg g^−1^ Se) and non-accumulators (<100 μg g^−1^ Se) (Brown and Shrift, 1982; Anderson, 1993). Selenium hyperaccumulation traits occur in a number of species across many families, particularly the Fabaceae, Brassicaceae and Asteraceae (El Mehdawi and Pilon-Smits, 2012). Notably, the genus *Astragalus* (Fabaceae) has 25 different taxa capable of hyperaccumulating Se (White, 2016), among which *Astragalus bisulcatus* and *A. racemosus* can accumulate Se at concentrations of ≤13 685 and ≤14 920 μg g^−1^, respectively, when growing in native soils (Sura-de Jong *et al.*, 2015). Brassicaceae species, such as *Stanleya pinnata* and *S. bipinnata*, are also notable hyperaccumulators able to accumulate 4000 and 2490 μg g^−1^ Se, respectively, when growing in soils containing 2–10 μg g^−1^ Se (Beath *et al.*, 1940; Galeas *et al.*, 2007). Other Se hyperaccumulators occurring in the western USA include species in the genera *Oonopsis*, *Xylorhiza* and *Symphyotrichum* (Asteraceae) (El Mehdawi *et al.*, 2014). In addition to these, Se hyperaccumulators are known from seleniferous soils in central Queensland (Australia), where *Neptunia amplexicaulis* (Fabaceae) was discovered to hyperaccumulate ≤4000 μg g^−1^ Se in natural conditions (Peterson and Butler, 1967) and ≤13 600 μg g^−1^ Se under experimental conditions (Harvey *et al.*, 2020). Selenium hyperaccumulation has probably evolved independently in these different taxa, in response to similar environmental pressures (Pilon-Smits, 2017).

Selenium is not recognized as an essential element for higher plants, but in low doses it exerts several beneficial effects by promoting plant growth and cellular antioxidant activity (Hartikainen, 2005). Furthermore, when accumulated at high concentrations in Se hyperaccumulators, this metalloid provides protection against herbivores and pathogens, in addition to allelopathic benefits against competing species (El Mehdawi and Pilon-Smits, 2012). The main chemical forms that plants are able to take up are selenate (SeO_4_^2−^), highly bioavailable in oxidizing conditions, and, to a lesser extent, selenite (SeO_3_^2−^), which dominates in reducing conditions (Dinh *et al.*, 2019). Plants can also absorb elemental Se (Se^0^) and organic Se species, but not selenides (Chauhan *et al.*, 2019) or colloidal elemental Se (White, 2016). In general, selenate transport occurs through sulphate transporters, whereas selenite is taken up via phosphate and silicon transporters or anion channels (Zhang *et al.*, 2014; Wang *et al.*, 2019). The main difference between hyperaccumulators and non-hyperaccumulators is that the latter seem unable to distinguish between Se and S, whereas the former can apparently do so and preferentially take up Se, resulting in higher Se:S ratios in their tissues compared with non-hyperaccumulating species (Cappa and Pilon-Smits, 2014; Harris *et al.*, 2014). The ability to discriminate between Se and S could be attributable to the existence of S/Se transporters with a greater specificity for Se over S (El Mehdawi *et al.*, 2015). In addition, the Se hyperaccumulators *A. racemosus*, *A. bisulcatus* and *S. pinnata* are reported significantly to overexpress genes related to root S/Se uptake (such as the SULTR genes), resulting in very high levels of both elements in their tissues (El Mehdawi *et al.*, 2015; Schiavon *et al.*, 2015; Wang *et al.*, 2019).

High concentrations of Se in plant tissues cause oxidative stress and protein folding disruption (Van Hoewyk, 2013). However, Se hyperaccumulators are known to convert most of the inorganic Se to organic forms, such as the amino acid selenocysteine (SeCys), and then either methylate these forms to reduce their toxicity further or decompose SeCys into alanine and elemental Se (Sors *et al.*, 2009; Pilon-Smits, 2017). Methyl-SeCys constitutes the main form of organic Se accumulated in the plant tissues (>80 %) of most hyperaccumulators (Freeman *et al.*, 2006), but other compounds can also occur. In *A. bisulcatus*, for example, Se can occur in the form of γ-glutamyl-methyl-SeCys in the seeds and in the form of Se^0^ in the roots, stems and flowers (Valdez Barillas *et al.*, 2012). In older leaves, lower concentrations (30 %) of inorganic Se (SeO_4_^2−^ and SeO_3_^2−^) have also been detected (Freeman *et al.*, 2006). *Stanleya pinnata*, besides accumulating Se as methyl-SeCys, has significant prevailing concentrations of selenocystathionine, SeCys and SeCystine, which are not known in *A. bisulcatus* (Freeman *et al.*, 2006; Pilon-Smits, 2017). Selenocystathionine has also been reported in *N. amplexicaulis* (Peterson and Butler, 1967), in addition to other organic forms, including methyl-SeCys and selenomethionine (Harvey *et al.*, 2020).

In *A. bisulcatus*, *S. pinnata* and *N. amplexicaulis*, the highest Se concentrations invariably occur in the young leaves and reproductive tissues, mainly because all these species actively remobilize Se from older to younger tissues via the phloem (Freeman *et al.*, 2006; Valdez Barillas *et al.*, 2012; Harvey *et al.*, 2020). Nevertheless, there are consistent differences in the Se sequestration between these species. Selenium was found to be localized in the trichomes of *A. bisulcatus*, and high Se concentrations were determined in the petals and sepals (Freeman *et al.*, 2006; Valdez Barillas *et al.*, 2012). In the root of *A. bisulcatus*, the highest Se concentrations were in the cortex and stele (Valdez Barillas *et al.*, 2012). In *S. pinnata*, Se accumulates predominantly at the margins of young leaves, probably inside the vacuoles of large epidermal cells, and within the tissues of young leaf petioles (Freeman *et al.*, 2006; Cappa *et al.*, 2015), whereas in the root Se appeared to be stored within specialized cell vacuoles (Amos *et al.*, 2012). In contrast in *A. bisulcatus*, the highest Se concentrations in reproductive tissues occur in the stamen and pistil (Valdez Barillas *et al.*, 2012). Finally, studies on *N. amplexicaulis* revealed preferential localization of Se inside the veins (inside phloem bundles) of the young leaves and in the primary and secondary pulvini. High Se concentrations were also detected within the reproductive tissues, including the peduncle of the flower, anthers and filaments, in the infructescence and in the tap root (Harvey *et al.*, 2020).

**Fig. 1.**
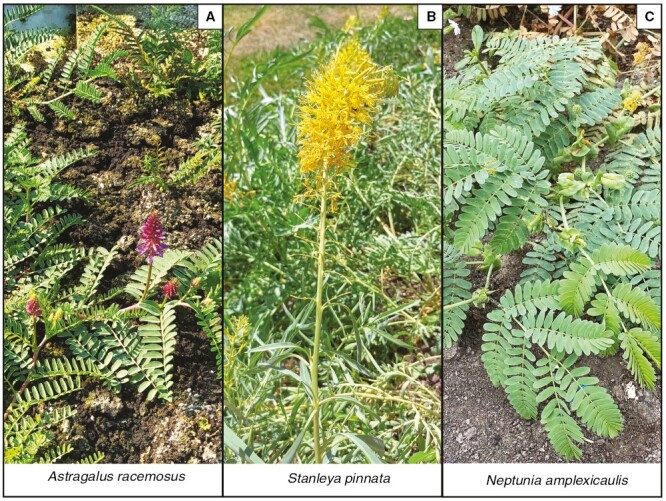
Mature flowering Se hyperaccumulator plants, *Astragalus racemosus* (A), *Stanleya pinnata* (B) and *Neptunia amplexicaulis* (C), growing in the glasshouse under controlled conditions (~6–8 months old).

*Astragalus bisulcatus* and *S. pinnata* are the most intensively studied Se hyperaccumulator model plant species and were previously subjected to synchrotron-based micro-X-ray fluorescence (µXRF) investigations to elucidate the distribution of Se and other elements, as described above. However, the used techniques were applied to freeze-dried samples, and this method is known to have the potential to introduce sample artefacts that affect elemental distribution and data interpretation (van der Ent *et al.*, 2018). In contrast, the present study uses the cutting-edge capabilities of the P06 beamline at the German Synchrotron (DESY, Hamburg, Germany) with fast detection to permit elemental mapping of *A. racemosus*, *S. pinnata* and *N. amplexicaulis* plant organs in their fresh hydrated state without any sample preparation artefacts. We aimed to determine whether the distributions of Se in the three key Se hyperaccumulating genera, *Astragalus*, *Stanleya* and *Neptunia* (Fig. 1), are similar or contrasting, in order to infer the physiological mechanisms that are responsible for Se hyperaccumulation. Comparison of these three species might provide insights into the influence of ecological/climatic factors vs. physiological similarities attributable to taxonomic placement. *Astragalus* and *Stanleya* occur in different families but have evolved hyperaccumulation in the same geographical areas in western North America, under the same selection pressures, whereas *Neptunia* evolved hyperaccumulation in Queensland, Australia. In contrast, *Astragalus* and *Neptunia* are both Fabaceae, whereas *Stanleya* belongs to the Brassicaceae. From this study, we expect to gain a better understanding of the strategies that phylogenetically distant Se hyperaccumulators evolved. This information will have ecological relevance because the hyperaccumulation of Se in specific organs or tissues and in specific chemical forms can influence the deterrence of herbivores, the trophic transfer of Se and changes in soil chemistry. It will also pave the way for further studies on Se hyperaccumulators aimed at using these species as a genetic resource for the development of staple crops biofortified in Se.

## MATERIALS AND METHODS

### Plant cultivation and selenium dosing conditions

Seeds of *N. amplexicaulis* were originally sourced from plants growing near Richmond, Queensland, Australia. The seeds were germinated by them piercing with a scalpel and leaving to soak in water for 24 h before being transferred to 0.3 L containers (three plants per pot) filled with standard potting mix. Seeds of *A. racemosus* were purchased from a specialized seed supply company in Colorado, USA (Prairie Moon Nursery, MN, USA) and likewise germinated and grown in standard potting mix. The plants were cultivated in a growth chamber illuminated with high-intensity LED lighting (500 μmol m^2^ s^−1^) at 25 °C and 70 % relative humidity (12 h–12 h light–dark) and were watered weekly with ~50 mL per pot of a solution containing 200 mg L^−1^ total Se. *Stanleya pinnata* seeds were purchased from the same supplier as noted above and the seeds were soaked in tap water for 12 h, then placed in 3-cm-diameter containers filled with 50 % river sand and 50 % turf and allowed to germinate in a growth chamber at temperature of 23 °C with 16 h–8 h light–dark, 70 % humidity and 500 μmol m^2^ s^−1^ light. Two-week-old plantlets were transferred into 20-cm-diameter pots (with two plants per pot) filled with 50 % river sand and 50 % turf and cultivated for 5 months in a glasshouse. Artificial lighting (TLED 42 W growing spectrum, Secret Jardin, Belgium) and heating assured a cycle of 16 h–8 h light–dark and a temperature of 20 °C. Each pot was fertilized with 2 g per pot of Osmocote^®^ 9 Month Slow-Release Fertiliser and watered weekly with 400 mL of 0.5 mm Se solution. In all treatements, Se was provided through an equimolar mixture of selenate and selenite. The choice of using two forms of Se simultaneously allowed a better simulation of the soil conditions in which both selenate and selenite are present and transform from one to the other depending on the soil redox potential.

### Histology and light microscopy of plant tissues

Leaf, petiole, stem and root cross-sections of *A. racemosus*, *S. pinnata* and *N. amplexicaulis* were dissected into 0.5 cm pieces. These were fixed with FAA solution (5 % v/v formaldehyde, 10 % v/v glacial acetic acid, 35 % v/v distilled water and 50 % v/v ethanol), and the samples were left in the solution for 24 h. Samples were dehydrated in a graded series of 25, 50 and 75 % (v/v) ethanol solutions. The samples were successivelyplaced in these solutions for 15, 30 and 60 min, respectively. Afterwards, the tissue pieces were washed in 100 % (v/v) ethanol three times (60 min each). The sections were immersed three times (10 min each) in pure Histolemon (CARLO ERBA Reagents S.r.l., Italy) before paraffin wax embedding (Sigma-Aldrich, St. Louis, MO, USA). To ensure paraffin penetration within the sample tissues, the sections were first immersed in liquid paraffin at 56 °C overnight under vacuum (−0.5 bar). The samples were then transferred into small containers filled with liquid paraffin and arranged in a vertical position. Paraffin cylinders were mounted on stubs and refrigerated at 4 °C for 10 min. Stubs were placed in a MR258 manual rotary microtome (Auxilab, Beriáin, Spain), and 10-µm-thick paraffin wax sections were collected. The sections were placed on microscope slides on a mixture of 80/20 % v/v water and albumin. Slides were then dried on a warm surface at 40 °C for 3 h. A solution of 1 % (v/v) sodium acetate and 0.1 % (v/v) Toluidine Blue was used for the staining, and the samples were soaked in the staining solution for 5 min., then rinsed with deionized water and left to dry on a warm surface at 40 °C overnight. Finally, the samples were immersed in Histolemon for 1 min. to remove paraffin. A coverglass was affixed to the surface using Canada balsam (Merck, Germany). The tissue samples were imaged on a Leica ICC50W microscope (Wetzlar, Germany) with a ×40 objective.

### Plant specimen preparation for synchrotron micro-X-ray fluorescence analysis

The potted live plants were transported to the PETRA III—Deutsches Elektronen-Synchrotron (DESY) facility (Hamburg, Germany) for analysis. Tissue samples (0.5-mm-thick pieces) from the petioles and leaf blades were cut from live plants immediately prior to µXRF 2D analysis using a stainless steel razor blade, using the dry knife method to avoid elemental displacement and losses (van der Ent *et al.*, 2018), and quickly mounted between two sheets of polypropylene 6 µm Ultralene® thin-film (Cole-Parmer, Vernon Hills, IL, USA), stretched over a plastic holder frame. This procedure prevented the samples from dehydrating and degrading during the analysis. For the µXRF-computed tomography measurements, the specimens were mounted inside Kapton capillaries and placed under a cryostream (operated at −160 °C) within 5 min. of excision, where they were kept for the duration of the measurement to minimize radiation damage.

### Synchrotron micro-X-ray fluorescence experiments

The X-ray fluorescence microscopy experiments were undertaken at PETRA III (Deutsches Elektronen-Synchrotron, DESY), a 6 GeV synchrotron radiation source, specifically at the hard X-ray microprobe experiment at the undulator beamline P06 (Boesenberg *et al.*, 2016). A combination of two-dimensional (2D) µXRF analysis of intact leaves, physical cross-sections of roots, stems, petioles and leaves, and µXRF-computed tomography (µXRF-CT) virtual slices of roots and petioles was used. The physical cross-sections have the advantage of minimizing self-absorption effects, whereas the virtual slices avoid effects of destructive sample preparation. All details on scan parameters are reported in Table 1. P06 is equipped with a cryogenically cooled double-crystal monochromator with Si (111) crystals. An ion chamber upstream of the sample is used to monitor the incoming flux, while a 500-µm-thick Si PIPS diode with 19-mm-diameter active area [PD300-500CB, Mirion Technologies (Canberra) GmbH, Germany] located downstream of the sample can be used to record the transmitted X-ray intensity in order to extract absorption data. Multiple X-ray fluorescence (XRF) detectors allow for the measurement of XRF data. The data presented in this manuscript were measured at an incident X-ray energy of 17 keV. A stack of beryllium compound refractive lenses (CRLs) (RXOPTICS, Germany) in combination with a corrective phase plate (Schropp *et al.*, 2019) was used to focus the X-ray beam down to 1 µm × 1 µm for 2D and 600 nm × 500 nm for three-dimensional (3D) measurements (horizon × vertical), resulting in a flux of ~1.5^10^ photons s^−1^ in the focus. Detection of XRF was performed by a four-element Vortex silicon drift detector (ME4, 350-µm-thick chip with 170 mm^2^ combined active area for all four elements, Hitachi High-Tech, USA) with Xspress 3 pulse processors (Quantum Detectors, UK) in 225° geometry for 2D and 270° for 3D measurements, with the forward direction of the X-ray beam defined as 0°. The tomographic measurements were conducted in single-slice tomography mode.

**Table 1.** Experimental and scanning parameters for the different synchrotron X-ray fluorescence microscopy experiments in this study.

Parameter	Figure 3	Figure 4	Figure 5	Figure 6	Figure 7
Species	*A. racemosus* *S. pinnata* *N. amplexicaulis*	*A. racemosus* *S. pinnata* *N. amplexicaulis*	*A. racemosus* *S. pinnata* *N. amplexicaulis*	*A. racemosus*	*S. pinnata*
Plant organ	Roots and stems	Roots and petioles	Leaves	Leaves	Leaf tip
Resolution (μm)	5, 8, 5	1, 0.75, 1, 1, 1, 1	7, 20, 6	10, 2	2, 4
Number of lateral steps	500 × 460 412 × 400 740 × 440	2400 × 180, 1600 × 180, 2400 × 180, 1200 × 180, 2250 × 360	1857 × 714 2700 × 2350 1333 × 833	1300 × 1000	1000 × 1450 500 × 150
Angles	–	1800, 1440, 720, 1800, 1440, 1440	–	–	–
Dwell time (ms)	10, 10, 5	10, 15, 10	5, 5, 5	5	4, 4
Total scan duration (min)	42, 31, 30, 34, 37, 13	749, 594, 152, 749, 750, 832	613, 758, 116, –, 99	117, 37	106, 39
Cryostream	No	Yes	No	No	No
Sample state	Fresh/hydrated	Frozen/hydrated	Fresh/hydrated	Fresh/hydrated	Fresh/hydrated
Sample preparation	Dry knife section	None	None	None	Dry knife section

### Bulk elemental analysis of plant tissues used during the synchrotron experiments

The plant material remaining after the synchrotron experiments were divided, stored in paper envelopes, and then dried in a drying oven at 60 °C for ≥48 h. Plant organs were ground to a fine powder (<200 µm) in an impact mill and weighed to 50 ± 5 mg in 15 mL polypropylene tubes. These samples were pre-digested for 16 h with 1 mL HNO_3_ (70 %) and 2 mL H_2_O_2_ (30 %), then digested in a block heater (DigiPREP MS, SCP SCIENCE, Baie-D’Urfe, Quebec, Canada) for a 180 min. programme (ramping up and holding at 95 °C). The digestates were then diluted to 10 mL with ultrapure water (Millipore 18.2 MΩ cm at 25 °C) and filtered through 0.45 µm syringe filters before analysis via inductively coupled plasma atomic emission spectroscopy (ICP-AES) using a Thermo Scientific iCAP PRO Duo X instrument (Waltham, MA, USA). The analysis was conducted on the old leaves and roots of all species, on the young leaves of *S. pinnata*, and on the stems of *N. amplexicaulis* and *S. pinnata*. The analysis was not performed on young leaves of *N. amplexicaulis* and *A. racemosus* or on stems of *A. racemosus* because the amount of material was not sufficient owing to the very small plants used for analysis (young seedlings).

### Data processing and statistics of the XRF data

The XRF data were processed using non-linear least-squares fitting as implemented in PyMCA (Solé *et al.*, 2007). In order to conduct the elemental calibration, various thin film deposited foils with XRF emission lines well spaced over the significant energy range were measured (Micromatter Technologies Inc., Canada). The elemental calibrations for other elements were calculated using the results of the foil measurements in combination with elemental parameters from *xraylib* (Schoonjans *et al.*, 2011). This produced a 32-bit .tiff, with pixel values corresponding to micrograms per centimetre squared areal density of the element in question. The figures were prepared in ImageJ (Schneider *et al.*, 2012) by changing the lookup table (LUT) to ‘Fire’, adjusting the maximum values and adding length scales. The tomographic data were aligned with tomographic consistency methods to correct for unwanted sample movement in horizontal direction. All datasets were reconstructed using a maximum-likelihood expectation-maximization (MLEM) algorithm (Bruyant, 2002). The tomograms shown were calibrated and quantified, apart from the self-absorption effects, which are limited for Se. To assess statistical differences in elemental concentration among plant organs, we used the non-parametric Kruskal–Wallis test followed by Dunn’s post hoc test.

## RESULTS

### Selenium and sulphur concentrations in plant organs by bulk analysis

The Se dose rates applied to the plants were sufficiently high to allow for the hyperaccumulation of Se in different organs of the three plant species in concentrations comparable or higher to those recorded in the Se hyperaccumulators growing in natural seleniferous soils (Beath *et al.*, 1940). *Astragalus racemosus* and *N. amplexicaulis* plants were dosed with an equal mixture of selenate and selenite (200 mg L^−1^ total Se). Based on the concentrations of Se in the old leaves, *A. racemosus* proved to be a stronger Se hyperaccumulator than *N. amplexicaulis* (Table 2), with a 28 500 μg g^−1^ Se in old leaves, which was 5.5-fold higher than the Se concentration in the old leaves of *N. amplexicaulis*. These values for *A. racemosus* are 2-fold higher than those reported in natural environments, where it can reach 14 920 μg g^−1^ Se (Knight and Beath, 1937), probably owing to the high dose rates used in the present research. Unlike *N. amplexicaulis* and *S. pinnata*, *A. racemosus* accumulated more Se in the old leaves than in the roots (~4.7-fold; Table 2). *Stanleya pinnata*, in contrast, accumulated Se preferentially in young leaves at concentrations 6.3-fold higher than in the old leaves. The Se concentrations in the stems was intermediate between values measured in roots and old leaves in *N. amplexicaulis*, whereas in *S. pinnata* stems and root the Se concentrations were comparable.

**Table 2.** Total Se and S concentrations (in micrograms per gram dry weight) and Se:S ratios in organs of *Astragalus racemosus*, *Stanleya pinnata* and *Neptunia amplexicaulis*. Data are the average ± SD of three biological replicates (*n* = 3). Letters indicate significant difference among plant organs of the same species after Kruskal–Wallis tests, followed by Dunn’s post hoc tests (*P* < 0.05).

Species	Plant organ	Se (μg g^−1^ dry weight)	S (μg g^−1^ dry weight)	Se:S
*A. racemosus*	Root	6100 ± 700^a^	2000 ± 200^a^	3.1 ± 0.4^a^
*A. racemosus*	Old leaves	28 500 ± 3600^b^	12 500 ± 3700^b^	2.4 ± 0.5^a^
*S. pinnata*	Root	4500 ± 900^a^	8900 ± 2100^a^	0.5 ± 0.2^a^
*S. pinnata*	Old leaves	1900 ± 800^b^	6800 ± 200^a^	0.3 ± 0.1^b^
*S. pinnata*	Young leaves	11 900 ± 3400^c^	13 600 ± 2200^b^	1.2 ± 0.2^c^
*S. pinnata*	Stems	4400 ± 100^a^	6400 ± 800^a^	0.7 ± 0.1^a^
*N. amplexicaulis*	Root	8300 ± 2000^a^	2700 ± 300^a^	3.0 ± 1.0^a^
*N. amplexicaulis*	Old leaves	5200 ± 900^b^	2600 ± 300^a^	2.0 ± 0.2^b^
*N. amplexicaulis*	Stems	6800 ± 400^a^	2190 ± 90^a^	3.1 ± 0.3^a^

The concentrations of S in the different organs of Se hyperaccumulators in most cases followed a trend similar to that of the accumulation of Se in the different organs (Table 2). *Astragalus racemosus* had higher S concetrations in the old leaves than in roots, whereas in *N. amplexicaulis* S accumulation decreased according to the following trend: roots > old leaves > stem. In *S. pinnata*, S concentration was higher in young leaves, whereas it accumulated in lower concentrations in other organs, such as the old leaves and the stem. The Se:S ratios were comparable between *A. racemosus* and *N. amplexicaulis*, and higher in the roots than in the old leaves. In *S. pinnata*, the Se:S ratio was lower compared with the other Se hyperaccumulators, and maximum in the young leaves. The total concentration of Ca, Fe, K, Mg, P and Fe in organs of *Astragalus racemosus*, *Stanleya pinnata* and *Neptunia amplexicaulis* are given in Table S1.

### Selenium in sections of roots and stems of *Astragalus*, *Stanleya* and *Neptunia* species

**Fig. 2.**
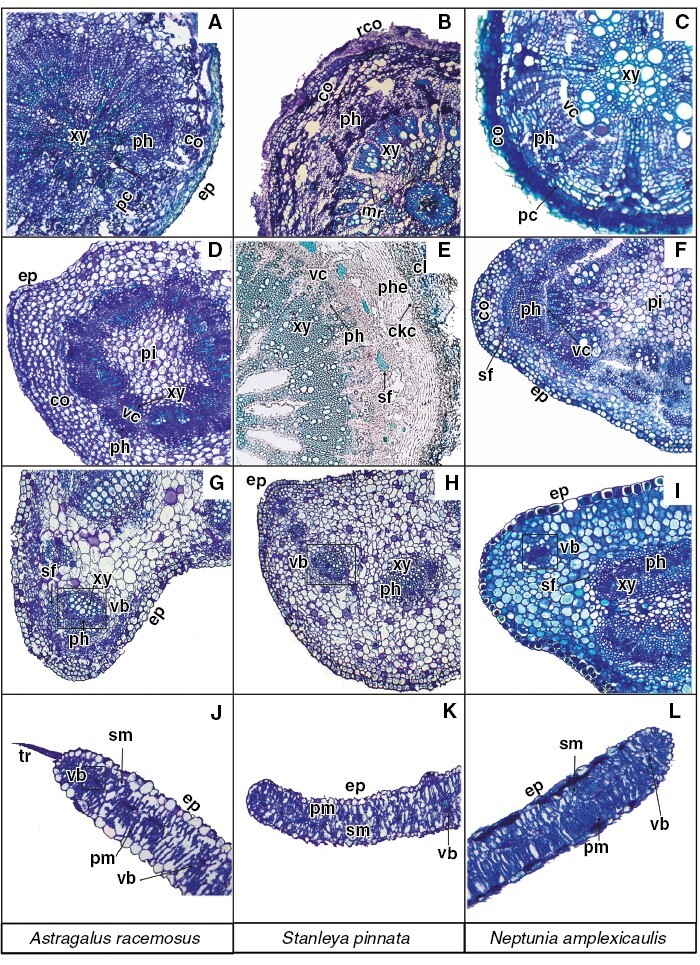
Toluidine Blue-stained histological sections of *Astragalus racemosus*, *Stanleya pinnata* and *Neptunia amplexicaulis*. (A–C) Root. (D–F) Stem. (G–I) Petiole. (J–I) Leaf. Abbreviations: ck, cork; ckc, cork cambium; co, cortex; ep, epidermis; mr, medullary rays; pc, pericycle; ph, phloem; phe, phelloderm; pi, pith; pm, palisade mesophyll; rco, residual cortex; sf, sclerenchyma fibres; sm, spongy mesophyll; tr, trichome; vb, vascular bundle; vc, vascular cambium; xy, xylem.

Figure 2 shows the anatomical features of histological cross-sections of the leaves, stems and roots of the studied Se hyperaccumulator species acquired after Toluidine Blue staining and used as a reference for interpretation of elemental localization in the μXRF data.

**Fig. 3.**
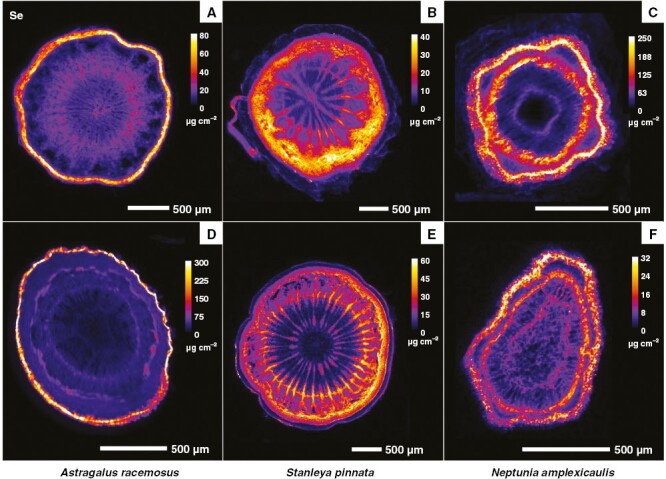
Synchrotron µXRF elemental maps showing the distribution of Se in fresh hydrated root (A–C) and stem (D–F) physical cross-sections of *Astragalus racemosus* (A, D), *Stanleya pinnata* (B, E) and *Neptunia amplexicaulis* (C, F).

**Fig. 4.**
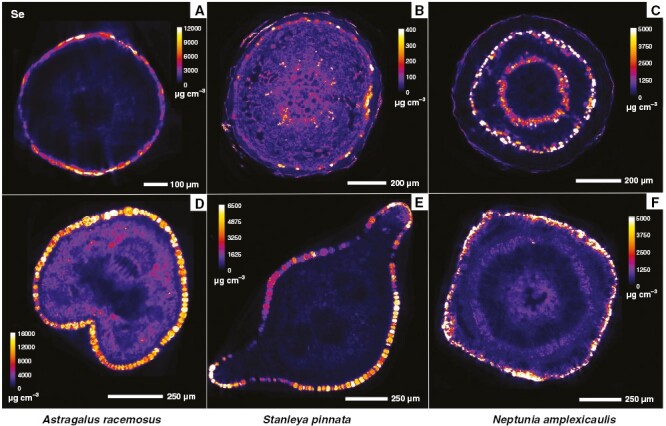
Synchrotron µXRF-CT elemental maps showing the distribution of Se in fresh hydrated roots (A–C) and petioles (D–F) in reconstructed virtual cross-sections of *Astragalus racemosus* (A, D), *Stanleya pinnata* (B, E) and *Neptunia amplexicaulis* (C, F).

Synchrotron µXRF elemental map and µXRF-CT reconstructions of *A. racemosus* roots showed localized regions of dense Se accumulation, which were the rhizodermal cortical cells and, to a lesser extent, the inner parenchymal pith tissues and phloem cells (Figs 3A and 4A). Conversely, in *S. pinnata* roots, Se accumulated principally in the cortical cylinder, specifically within the parenchymal cells of the sub-peridermal region (Figs 3B and 4B). In addition, the pith rays, the vascular cambium and the phloem cells had high Se too. In *N. amplexicaulis* roots, Se distribution was mainly restricted to the vascular cambium, secondary phloem vessels and cells of the cortical cylinder localized in the sub-peridermal region (Figs 3E and 4C). The patterns of Se distribution in roots of the three species was confirmed via XFM-CT analysis (Fig. 4A–C), which revealed similar distributions, but without some evident sample preparation artefacts, as explained in the Discussion section later.

The distribution of Se in the stems differed substantially among *A. racemosus*, *S. pinnata* and *N. amplexicaulis*. The distribution of Se in *A. racemosus* stems revealed almost exclusive occurrence of Se in the epidermal cell layer (Fig. 3D). The vascular tissues in the mid-shaft and the parenchymal cells in the cortical cylinder were not sites of Se accumulation in this species. In *S. pinnata* stem sections, Se occurred mainly in the pith rays, vascular cambium and parenchymal cells of the sub-epidermal cortical layer (Fig. 3C). The epidermal and phloem cells were also Se enriched, but the concentrations were lower than in the previous discussed tissues. In *N. amplexicaulis*, Se occurred mainly in the cells of the sub-epidermal cortical layer, in vascular cambium and in secondary phloem cells (Fig. 3F). The pith rays were also Se enriched, but lesser so with respect to *S. pinnata* stems. Cells containing moderate Se concentrations at the peripheral area of the inner parenchymal pith tissue were evident (Fig. 3F). Despite differences in Se distribution in roots and stems, the major commonality observed among the three Se hyperaccumulator species was the general absence of Se in the xylem cells of the roots (Figs 3A–C and 4A–C) and stems (Fig. 3D–F).

### Selenium in petiole and whole leaf sections of *Astragalus*, *Stanleya* and *Neptunia* species

**Fig. 5.**
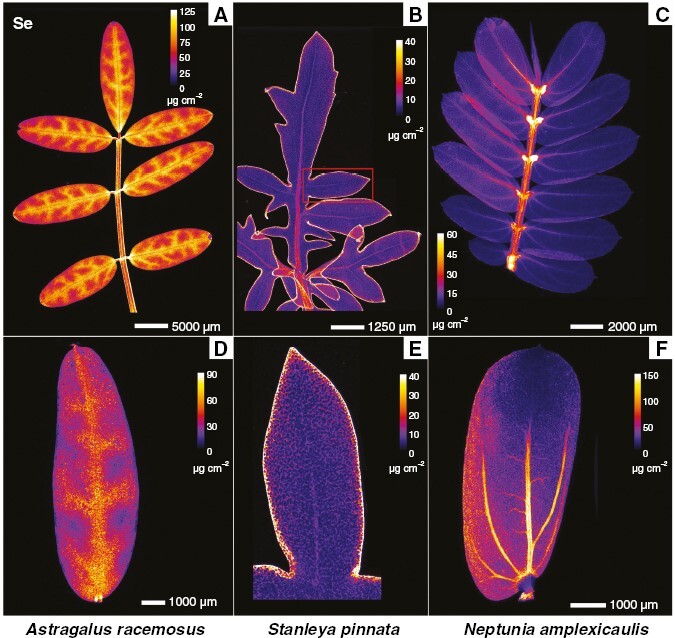
Synchrotron µXRF elemental maps showing the distribution of Se in fresh hydrated leaves of *Astragalus racemosus* (A, D), *Stanleya pinnata* (B, E) and *Neptunia amplexicaulis* (C, F). (D–F) For *S. pinnata*, E is an excerpt from B, whereas D and F are separate high-resolution scans.

**Fig. 6.**
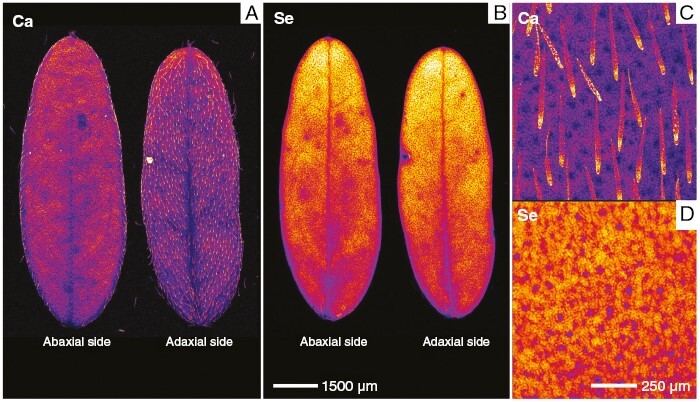
Synchrotron µXRF elemental maps showing the distribution Ca (A) and Se (B) in fresh hydrated leaves of *Astragalus racemosus*. (C, D) Detail of Ca (C) and Se (D) distribution on the adaxial side.

The analysis of Se distribution in the petioles using the synchrotron µXRF-CT reconstructed virtual sections revealed, for all Se hyperaccumulators, high Se concentrations in the epidermal cells and an absence of Se in the xylem (Fig. 4D–F). In *A. racemosus*, Se accumulated in lower concentrations in the parenchymal cells (Fig. 4D). The analysis of the whole *A. racemosus* leaves showed that Se was concentrated primarily in the pulvini of the leaves and in the cells surrounding the leaf vascular bundles, but not in the leaf margins (Fig. 5C). The high-resolution scan of an *Astragalus* individual leaflet revealed that Se accumulated in the vacuolar compartment (Fig. 5D). Individual leaflets of *A. racemosus* were also analysed on their abaxial and adaxial sides (Fig. 6) to assess whether Se might be localized in the trichomes, as reported in a previous study (Freeman *et al.*, 2006). The relatively high energy of Se fluorescence (11.2 keV) signalmeans that the signal penetrated through the entire thickness of the leaf, whereas for Ca fluorescence (3.6 keV) only the signal from the surface can be detected. This explains why the Ca-rich trichomes were clearly visible in the abaxial leaf (the surface facing the detector, Fig. 6A, C), whereas the Se maps of both leaf sides are nearly identical. Consequently, the present analysis clearly shows that Se does not localize in the trichomes, but rather occurs in the leaf parenchyma cells in large vacuoles (Fig. 6).

**Fig. 7.**
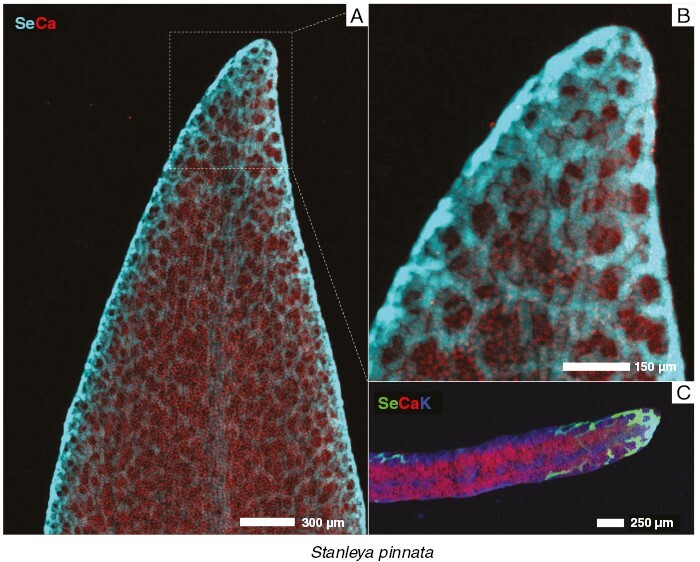
Synchrotron µXRF elemental maps showing the distribution of Se, Ca and K in a fresh hydrated leaf tip of *Stanleya pinnata*. (A) Whole leaf tip. (B) Details of the tip. (C) Physical cross-section of the leaf tip.

In *S. pinnata*, Se accumulates mostly in the young leaves in comparison to the old leaves. In old and young leaves, Se localizes predominantly along the leaf margins, with some enrichment in the central vascular bundle (Fig. 5B). A similar distribution of Se in the marginal area of the leaf was reported by Freeman *et al.* (2006), but the resolution of their scans was not sufficiently high to resolve the cellular-level detail shown here. In the present study, a high-resolution scan of the tip of one of the leaf lobes, in addition to a physical cross-section of a leaf, shows that Se occurrs principally in the apoplastic space across the leaf and most densely along the foliar margins and at the tip (Fig. 7).

The analysis of a full *N. amplexicaulis* leaf indicated that Se was concentrated primarily in the pulvini of the leaves as also observed for *A. bisulcatus* (Fig. 5C). From the single leaflet scan, it emerges that Se accumulates within the vascular bundles (Fig. 5F). These patterns were confirmed by earlier studies using laboratory and synchrotron-based µXRF analyses (Harvey *et al.*, 2020), during which Se in leaves of *N. amplexicaulis* was found to be highly concentrated in the vascular bundles and specifically in the phloem vessels. Furthermore, at the entire leaf scale, Se was clearly most accumulated in the youngest leaflets, in addition to being highly concentrated in the pulvini (Fig. 5C, F).

### Methodological observations: physical sections vs. virtual sectioning by µXRF-CT

The physical root sections (Fig. 3) have a thicker diameter (600–1000 µm), roughly twice that of the tomography root sections (400–500 µm). This is because very thin roots are extremely difficult to cut but are necessary to minimize X-ray self-absorption during µXRF-CT analysis. The physical and virtual sections of *A. racemosus* and *N. amplexicaulis* are similar, but those of *S. pinnata* are different, with the physical section having prominent medullary rays that are absent from the virtual section (which develop during root maturation). In the physical sections of *A. racemosus* and *N. amplexicaulis*, smearing of Se is evident, whereas features in the virtual sections are well defined, especially Se enrichment in cortex of *A. racemosus* (with the inner region markedly devoid of Se), and discrete phloem bundles in *N. amplexicaulis*. Stems were sectioned physically but not subjected to µXRF-CT because their thickness would cause significant self-absorption effects. Instead, the much thinner petioles were used for the µXRF-CT.

## DISCUSSION

Visualizing element distribution in plants is essential for understanding the physiological functioning of a plant and, in the case of Se hyperaccumulators, this approach allows for highlighting distinct biopathways of Se sequestration in different species. In X-ray fluorescence-based approaches, elements are detected based upon their characteristic fluorescent X-rays, and this unique approach provides *in situ* information (Kopittke *et al.*, 2018; van der Ent *et al.*, 2018) in biological conditions of the living plant. Nowadays, extremely bright X-ray sources at third-generation synchrotrons and ultra-fast detector systems make it feasible to analyse live hydrated plant tissues with minimum to no sample preparation at ambient temperature and pressure (Kopittke *et al.*, 2020). Studies that use these facilities have revolutionized the investigation of the metallome in live plant and hydrate tissues, including entire plants (Blamey *et al.*, 2018; Doolette *et al.*, 2018). Owing to the penetrating nature of X-rays, the elemental distribution of an intact leaf (or other intact plant organs) is projected onto a single plane. Consequently, to obtain information on the internal distribution of elements within plant organs, destructive sample preparation methods, such as sectioning, are required. To overcome this problem, XµXRF-CT enables virtual sectioning of a sample, thereby entirely avoiding artefacts arising from destructive sample preparation (Spiers *et al.*, 2022). In this study, we used µXRF-CT to localize Se in hydrated organs of three Se hyperaccumulators, as a different approach to previous studies using 2D modalities for Se visualization in plants (Harvey *et al.*, 2020).

We do not know whether Se hyperaccumulation arose sequentially in hypertolerant plants or whether Se hyperaccumulation and hypertolerance evolved simultaneously (Cappa and Pilon-Smits, 2014). Also, we do not know whether the trait of Se hyperaccumulation evolved independently in different species under similar or distinct selective pressures. This investigation sought to determine, via elemental distribution, whether Se hyperaccumulation in the three key genera of hyperaccumulators differs in their underlying mechanisms or whether these mechanisms might be convergent. Our results show that the distribution of Se is, to a certain level, common across all analysed species, but in addition underline the existence of some relevant differences indicating that distinct Se handling and tolerance systems likely evolved in *Astragalus*, *Stanleya* and *Neptunia* (Fig. 1).

The similarities between the three genera mainly relate to the preferential concentration of Se in the youngest plant organs and tissues, in particular young leaves. This has been observed recently in *N. amplexicaulis*, with old leaves containing ~1/10th the Se of young leaves (Harvey *et al.*, 2020). Concentrating Se in developing tissues is probably a common defence strategy activated in the Se hyperaccumulators, because the young parts of a plant are generally more vulnerable to attacks by herbivores and pathogens, and their high Se content would act as a deterrent to these organisms. Another commonality evident between Se hyperaccumulators is the epidermal sequestration of Se in leaves and petioles, both of which are targets of several predators (Freeman *et al.*, 2006). In these organs, Se is localized within large vacuoles. The reason why Se accumulates largely in this tissue is that epidermal cell layers are important in organ protection, are susceptible to biotic stress and can play some defensive roles (Freeman *et al.*, 2006). Selenium is stored in the vacuole because this compartment is less metabolically active than others (e.g. cytosol, mitochondria and plastids), thus Se will not cause oxidative stress to the cell itself. *Neptunia amplexicaulis* is unique, with a remarkable localization of Se also in its phloem tracts (Fig. 5F), whereas *S. pinnata* is distinctive in Se distribution in the apoplastic space, especially along the foliar margins (Fig. 5E).

In line with previous findings (Alford *et al.*, 2012), our study confirmed that Se is localized in large cells of the leaf blade, especially near the vascular bundles, and absent in the trichomes of *A. racemosus* (Fig. 6). This is not true for other hyperaccumulating *Astragalus* species (Pickering *et al.*, 2003); in fact, Freeman *et al.* (2006) found high Se concentrations in the trichomes of *A. bisulcatus*. Pushie *et al.* (2014) posited that these differences might be attributed to different culture methods used (commercial soil mix or hydroponics, respectively) or to the collection location (Fort Collins, CO, USA or Big Hollow, WY, USA, and Saskatoon, Saskatchewan, Canada, respectively) instead of this being species specific.

Differences in Se distribution are also evident in roots and stems of the three taxa, although a feature in common is that Se does not localize into the xylem but is evidently present in the phloem. Likewise, the lack of Se localization in the xylem was distinctly evident in the petioles of the three species (Fig. 4D–F). Consistent with the analysis of Se concentration in different organs, we hypothesize that Se is redistributed in these plants through the phloem from old leaves to developing leaves and to the roots. *Stanleya pinnata* and *N. amplexicaulis* have high Se accumulation in the peripheral region of the cortex, whereas *A. racemosus* accumulates large amounts of Se in the integument, in the root and in the stem (Fig. 4A–C). Given that the accumulation of metals/metalloids in the cortex periphery is believed to serve as a barrier for their radial transfer to the vascular bundles (Vaculik *et al.*, 2012), the absence of Se in this region could be associated with high Se translocation to and accumulation in the old leaves of *A. racemosus*. Interestingly, and never reported previously, *S. pinnata* has very high Se accumulation in the pith rays both in the root and in the stem, which means that the species presumably uses pith rays for the transport of Se throughout the plant but can also store Se in their cells as a protective mechanism.

In conclusion, this study has revealed broad patterns of Se distribution at organ, tissue and cellular levels. Fundamental similarities among the three hyperaccumulators were shown (i.e. maximum Se accumulation in the young emerging leaves, preferential localization of Se in the foliar apoplastic space in *Astragalus* and *Stanleya*, and an absence of Se in xylem vessels of roots, stems and petioles). Some important dissimilarities among the three analysed species, notably phloem localization of Se in *Neptunia*, support the thesis that Se hyperaccumulation has evolved multiple times under similar environmental pressures (seleniferous soils in a hot semi-arid climate) in different locations (USA vs. Australia). The fact that different Se-handling mechanisms evolved in Se hyperaccumulators finds further support in the major differences in the chemical speciation of Se in the three species, with methyl-selenocysteine in *Astragalus* and *Stanleya* and selenocystathionine in *Neptunia*. Interestingly, there were more similarities between *Astragalus* and *Stanleya*, although they are from completely different plant families, with respect to those detected between the two Fabaceae species *Astragalus* and *Neptunia* (although the latter does have in common that Se is concentrated in the pulvini).

This study has demonstrated how powerful synchrotron µXRF methods are for elucidating the distribution and, ultimately, revealing the mechanisms underlying Se hyperaccumulation. In future studies, synchrotron X-ray absorption spectroscopy (XAS)may be used to quantitatively determine Se chemical speciation , iin the different species under investigation. This application of XAS allows for a more comprehensive identification of potential variations in Se chemical forms and can provide further insights into the diverse mechanisms evolved in the various plant species in Se uptake, assimilation and storage.

The next frontier in our understanding of Se hyperaccumulation is to increase the scale of magnification to probe the subcellular distribution of Se in these species. Furthermore, future studies could focus on the investigation of closely related hyperaccumulating vs. non-accumulating species pairs. This approach will be particularly suitable for *Stanleya* because within this species Se hyperaccumulation is population specific. Ultimately, detailed insights into Se physiology are expected to be gleaned from transformation models in which key Se metabolism genes are knocked out.

## SUPPLEMENTARY DATA

Supplementary data are available at *Annals of Botany* online and consist of the following.

Table S1: total concentration of Ca, Fe, K, Mg, P and Fe in organs of *Astragalus racemosus*, *Stanleya pinnata* and *Neptunia amplexicaulis*.

mcad110_suppl_Supplementary_Table

## Data Availability

The data that support this study will be shared upon reasonable request to the corresponding author.
