# Supplementary material for: Differences and similarities in selenium biopathways in Astragalus, Neptunia (Fabaceae) and Stanleya (Brassicaceae) hyperaccumulators
Source: Ann Bot. 2023 Aug 21;132(2):349–61. doi: 10.1093/aob/mcad110 (PMC10583200; doi:10.1093/aob/mcad110)
Supplement: mcad110_suppl_Supplementary_Table [file mcad110_suppl_supplementary_table.docx]

SUPPORTING INFORMATION

**Differences and similarities in selenium biopathways in *Astragalus, Neptunia* (Fabaceae) and *Stanleya* (Brassicaceae) hyperaccumulators**

Antony van der Ent^1,2,3*^, Mirko Salinitro^4*^, Dennis Brueckner^5^, Kathryn M. Spiers^5^,

Sofia Montanari^4^, Annalisa Tassoni^4^, Michela Schiavon^6^

^1^Laboratory of Genetics, Wageningen University and Research, The Netherlands.

^2^Centre for Mined Land Rehabilitation, Sustainable Minerals Institute,

The University of Queensland, Queensland, Australia.

^3^Laboratoire Sols et Environnement, INRAE, Université de Lorraine, France.

^4^Department of Biological Geological and Environmental Sciences, University of Bologna, Italy.

^5^Deutsches Elektronen-Synchrotron DESY, Germany.

^6^Department of Agricultural, Forest and Food Sciences (DISAFA), University of Turin, Italy.

*Corresponding authors: antony.vanderent@wur.nl, mirko.salinitro2@unibo.it

**Supplementary table S1**. Total concentration of Ca, Fe, K, Mg, P, and Fe in organs of *Astragalus racemosus, Stanleya pinnata* and *Neptunia amplexicaulis*. Concentrations are expressed in μg g^-1^ DW and are the average ± SD of three biological replicates (n=3).

| **Species** | **Part** | **Ca** | **Fe** | **K** | **Mg** | **P** | **Zn** |
| --- | --- | --- | --- | --- | --- | --- | --- |
| *A. racemosus* | Root | 6900 ± 3400 | 110 ± 30 | 25,500 ± 3700 | 1700 ± 100 | 7100 ± 900 | 76 ± 5 |
| *A. racemosus* | Old leaves | 18,400 ± 3600 | 60 ± 20 | 28,200 ± 3000 | 8800 ± 1000 | 4300 ± 400 | 26 ± 5 |
| *N. amplexicaulis* | Root | 9730 ± 60 | 210 ± 30 | 11,900 ± 400 | 2900 ± 100 | 3900 ± 200 | 15 ± 1 |
| *N. amplexicaulis* | Old leaves | 7700 ± 400 | 51 ± 5 | 20,600 ± 1000 | 2300 ± 100 | 3300 ± 300 | 21 ± 4 |
| *N. amplexicaulis* | Stems | 7100 ± 600 | 52 ± 2 | 14,800 ± 1300 | 2710 ± 90 | 6100 ± 620 | 14 ± 9 |
| *S. pinnata* | Root | 9500 ± 700 | 52 ± 1 | 28,400 ± 4100 | 4000 ± 400 | 5800 ± 200 | 37 ± 7 |
| *S. pinnata* | Stems | 8500 ± 500 | 40 ± 4 | 31,200 ± 600 | 4600± 200 | 5600 ± 200 | 61 ± 7 |
| *S. pinnata* | Old leaves | 52,600 ± 1400 | 52 ± 4 | 37,200 ± 1900 | 7600 ± 1000 | 4200 ± 500 | 180 ± 40 |
| *S. pinnata* | Young leaves | 24,300 ± 9400 | 74 ± 6 | 33,600± 1700 | 3800 ± 1400 | 7200 ± 1000 | 140 ± 20 |
